# Spatial analysis of the geographical distribution of thyroid cancer cases from the first-round thyroid ultrasound examination in Fukushima Prefecture

**DOI:** 10.1038/s41598-018-35971-7

**Published:** 2018-12-05

**Authors:** Tomoki Nakaya, Kunihiko Takahashi, Hideto Takahashi, Seiji Yasumura, Tetsuya Ohira, Hitoshi Ohto, Akira Ohtsuru, Sanae Midorikawa, Shinichi Suzuki, Hiroki Shimura, Shunichi Yamashita, Koichi Tanigawa, Kenji Kamiya

**Affiliations:** 1 Graduate School of Environmental Studies, Tohoku University, Aoba-ku, Sendai-city, Miyagi 980-0845 Japan; 2 Department of Biostatistics, Nagoya University Graduate School of Medicine, Showa-ku, Nagoya-city, Aichi 466-8550 Japan; 3 National Institute of Public Health, Wako-city, Saitama 351-0197 Japan; 4 Radiation Medical Science Center for the Fukushima Health Management Survey, Fukushima Medical University, Fukushima-city, Fukushima 960-1295 Japan; 5 Department of Public Health, School of Medicine, Fukushima Medical University, Fukushima-city, Fukushima 960-1295 Japan; 6 Department of Epidemiology, School of Medicine, Fukushima Medical University, Fukushima-city, Fukushima 960-1295 Japan; 7 Department of Radiation Health Management, School of Medicine, Fukushima Medical University, Fukushima-city, Fukushima 960-1295 Japan; 8 Department of Thyroid and Endocrinology, Fukushima Medical University, Fukushima-city, Fukushima, 960-1295 Japan; 9 Department of Laboratory Medicine, School of Medicine, Fukushima Medical University, Fukushima-city, Fukushima 960-1295 Japan; 10 Department of Radiation Medical Sciences, Atomic Bomb Disease Institute, Nagasaki University, Nagasaki-city, Nagasaki 852-8523 Japan; 11 Research Institute for Radiation Biology and Medicine, Hiroshima University, Minami-ku, Hiroshima-city, Hiroshima 734-5844 Japan

## Abstract

Following the Fukushima Daiichi Nuclear Power Plant (FNPP) accident on 11 March 2011, there have been concerns regarding the health impacts of the ensuing radioactive environmental contamination, which was spatially heterogeneous. This study aimed to assess the geographical variability of thyroid cancer prevalence among children and adolescents in Fukushima Prefecture. We computed the sex- and age-standardised prevalence ratio using 115 diagnosed or suspected thyroid cancer cases among approximately 300,000 examinees at the first-round ultrasound examination during 2011–2015 from 59 municipalities in the prefecture, under the Fukushima Health Management Survey. We applied flexibly shaped spatial scan statistics and the maximised excess events test on the dataset to detect locally anomalous high-prevalence regions. We also conducted Poisson regression with selected regional indicators. Furthermore, approximately 200 examinees showed positive ultrasound examination results but did not undergo confirmatory testing; thus, we employed simulation-based sensitivity tests to evaluate the possible effect of such undiagnosed cases in the statistical analysis. In conclusion, this study found no significant spatial anomalies/clusters or geographic trends of thyroid cancer prevalence among the ultrasound examinees, indicating that the thyroid cancer cases detected are unlikely to be attributable to regional factors, including radiation exposure resulting from the FNPP accident.

## Introduction

The accident on 11 March 2011 at the Fukushima Daiichi Nuclear Power Plant (FNPP), triggered by the tsunami waves following the Great East Japan Earthquake, raised serious concerns about the health impact of radioactive materials released into the atmosphere from the FNPP. Possible risks of radiation-induced thyroid cancer among children in the radioactively contaminated areas have gained attention, due to the finding of a marked excess of this cancer type among European children who lived in areas affected by radioactive iodine released during the Chernobyl Nuclear Power Plant (CNPP) accident on 26 April 1986^1^. However, studies conducted in Japan just after the FNPP accident to estimate thyroid exposure from radioactive iodine released from the FNPP^2–4^ reported much lower thyroid doses among the evacuees, compared to the CNPP accident. Thus, radiation-induced excess health risks were not expected to be observable with exposure to such low doses^5^.

In October 2011, the Fukushima Prefectural government started an ultrasound-based examination programme for thyroid cancer (thyroid ultrasound examination: TUE) for all residents aged 18 years or younger who were living in Fukushima Prefecture at the time of the FNPP accident; this initiative was part of a larger programme to investigate the potential health impact of the Fukushima accident^6,7^. The period of the first round of TUE, from 2011 to 2014, was decided based on the finding that cases of cancer among children after the CNPP accident were detected four or five years later. Since radiation-related thyroid cancer would not be expected to develop during this period, the first round of TUE would give a measure of the baseline thyroid cancer prevalence for evaluation in subsequent rounds of examination in the same target population.

In the first round of TUE from 2011 to 2015 (including one extended year), 116 thyroid cancer cases with malignancy or strong suspicion of malignancy based on fine needle aspiration cytology were detected from approximately 300,000 children and adolescents. This larger-than-expected number of cases incited the suspicion of an ‘epidemic’ of early radiation-induced thyroid cancer^8^. However, since such a large-scale ultrasound examination targeting children and adolescents had never been performed in Japan, the number of detected cases from the TUE is not comparable to the expectation based on conventional statistics, such as cancer registry data, due to the different systems of case examination^9,10^.

Several studies have sought to statistically ascertain if a higher risk of thyroid cancer existed in radiation-contaminated areas within the prefecture. Tsuda *et al*.^8^ aggregated 59 municipalities in Fukushima Prefecture into 9 districts and compared the regional prevalence rates among them. They reported excesses of thyroid cancer cases in the central middle district (odds ratio to the reference region, 2.6; 95% confidence interval [CI], 0.99–7.0) suggesting that the geographical excess was induced by exposure of the residents to radioactive contamination within the environment. However, several studies pointed out that the identified regional difference in prevalence was inconsistent with the distribution of radioactive contamination and that there was no statistical support to the difference in prevalence between areas with different levels of radioactive contamination^10–12^. Ohira *et al*.^13^ constructed five areas from 59 municipalities according to the estimated degrees of external exposure to radiation among the TUE participants and found no significant differences in prevalence among them; compared to the area with the lowest dose, the highest age-sex adjusted odds ratio was in the area with the second highest dose (odds ratio, 1.44; 95% CI, 0.75–2.75) while the area with the highest dose had an odds ratio of 0.95 (95% CI, 0.48–1.88). It is noteworthy that the areas with the higher estimated level of external exposure to radiation were not those located around the FNPP but were in the northern middle region of the prefecture^13^; this could be possibly due to the movements of evacuees and radioactive plumes after the FNPP accident. However, due to the high degree of uncertainty in the estimation of internal dose from radioactive iodine-131, which has a short half-life of 8 days^1,4^, the distribution of internal exposure dose could differ from that of the external dose.

We identified three problems that needed to be overcome in these previous studies. Firstly, the results of the regional prevalence comparisons were dependent on the nature of the areal aggregation of municipalities. Thus, there remained the possibility of another way of areal aggregation to identify areas with significantly elevated risks. Such dependency of any spatial analysis on the geographic units used is known as the modifiable areal unit problem^14^. It is important to recognise that there are many possibilities in constructing regional units even if the basic unit of the municipality is given. It is also important to note that repeating comparisons of regional prevalence rates with a reference value increases the number of false positive risks. Hence, some significant difference would be detected at a certain alpha level of testing even if the null hypothesis is true. This is known as the multiple testing problem and requires proper adjustments of the significance level of the test^15^.

Secondly, there is potential bias caused by the fact that the ultrasound examination programme comprised two phases: primary evaluation using ultrasound and confirmatory testing^16^. Previously, during the primary examinations, the prevalence was evaluated as the number of diagnosed cases among examinees. This approach could be justified if it is assumed that no difference in thyroid cancer prevalence exists between examinees and non-examinees. However, not all positive examinees at the primary examination accepted confirmatory testing. Since the acceptance rate of the confirmatory testing among positive examinees varied geographically^8^, it is desirable to assess the possible impacts of undiagnosed cases in the statistical analysis.

Thirdly, although other risk factors could influence the prevalence of thyroid cancer, no analysis assessed geographic associations with other regional indicators besides radiation dose in Fukushima Prefecture^1,7^. Apart from ionising radiation exposure (including radioactive fallout, and natural background radiation), several geographic risk factors of thyroid cancer are recognised. The most well-known is the “inland mountain”, measured by altitude or rurality indices. Several parts of Europe, like the Alpine regions of Italy and the mountainous part of Wales in the UK, were characterised by iodine deficiency disorders and goitre endemicity^17,18^. These regions correspond to areas with high thyroid cancer morbidity and mortality. Other factors include areal indicators of socio-economic status (SES), including average income or racial components^19,20^. However, most ecological studies on the association between thyroid cancer and geographic factors, other than radiation, have not focused on thyroid cancer among children and adolescents.

To overcome these problems, this study aimed to assess the geographical variability in thyroid cancer prevalence obtained from the final results of the first round of TUE in Fukushima Prefecture among children and adolescents. By using spatial epidemiological methods, a basis is provided for evaluating the data that will be obtained from further rounds of TUE. More specifically, we employed spatial cluster detection methods and ecological regression models to identify: (1) whether any areas have excess risk/prevalence of thyroid cancer, and (2) whether any geographic indicator is associated with the distribution of thyroid cancer prevalence, with (3) sensitivity analysis to consider the possible impact of undiagnosed positive examinees on the results of the statistical analysis, at the primary ultrasound examination.

## Results

### Detecting regions with locally elevated risk

**Figure 1:**
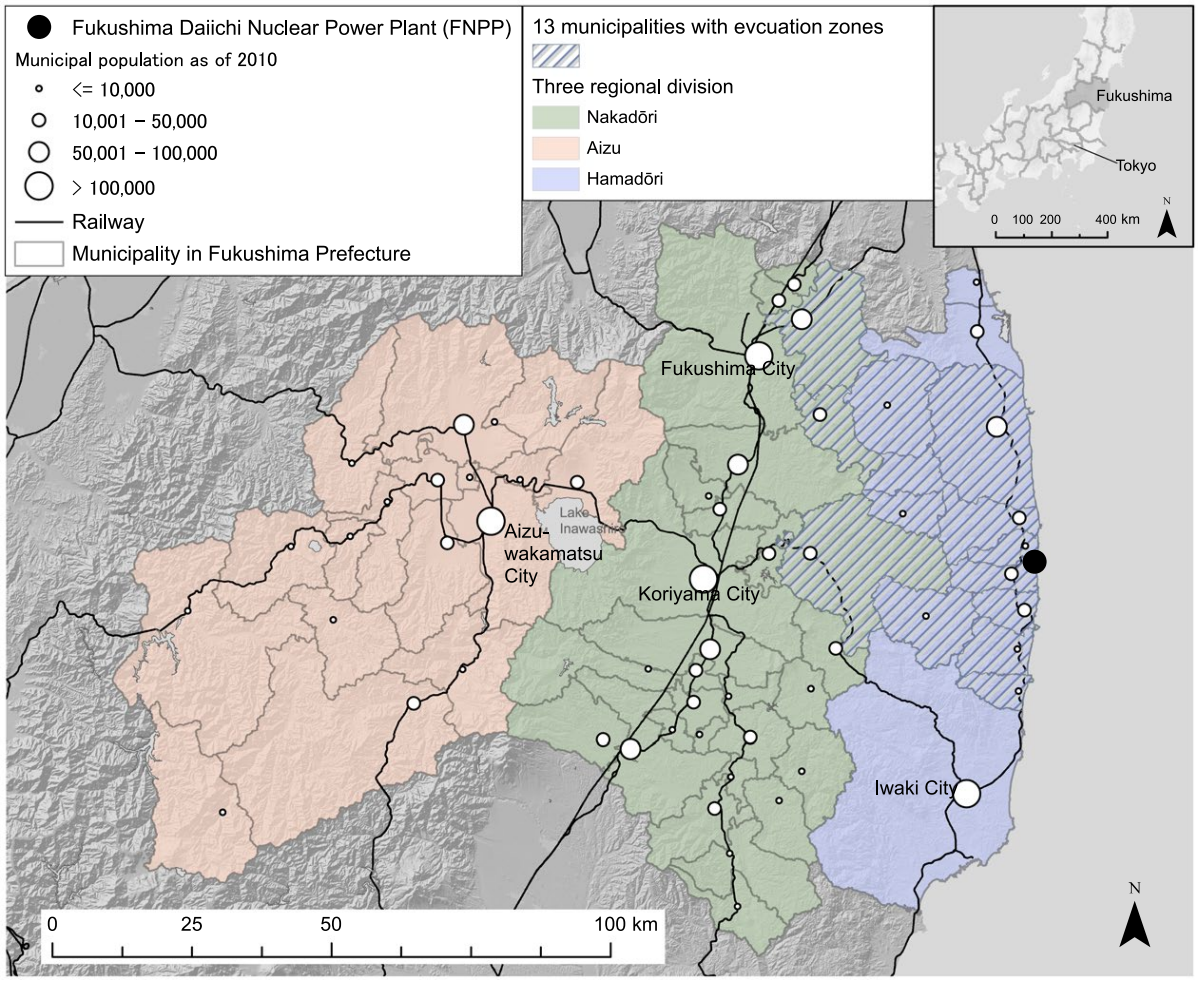
Study area, Fukushima Prefecture, Japan. Circles representing municipal census population as of 2010 were drawn at municipal town hall locations. The map in this figure was created with ArcGIS version 10.5 (http://desktop.arcgis.com/).

Fukushima Prefecture, the third largest Japanese prefecture, is located in northern Japan, and has an east to west length of about 165 km and a north to south length of 132 km (Fig. 1). The eastern part, Hamadōri, has its coastal plain facing the Pacific Ocean where the FNPP is located, approximately centrally on the coast of the prefecture. The middle part, Nakadōri, is the most densely populated area. The western part, Aizu, is mostly mountainous and the least inhabited. The prefecture comprises 59 municipalities that are the spatial units used in this study.

**Figure 2:**
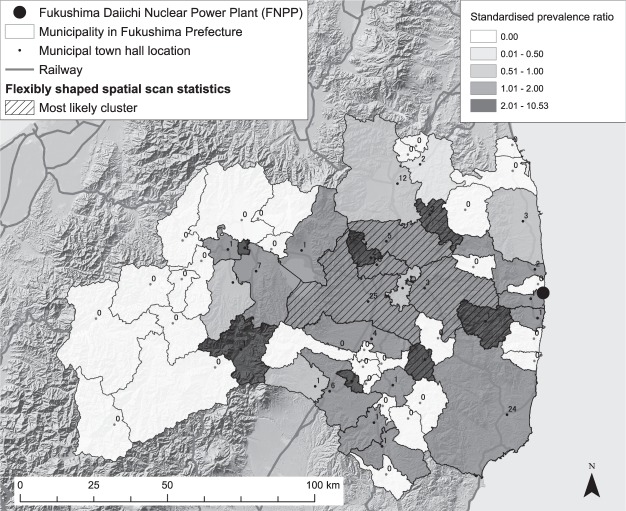
Standardised prevalence ratio and the most likely cluster of childhood and adolescent thyroid cancer cases derived from flexibly shaped spatial scan statistics in Fukushima Prefecture, Japan. The numbers near municipal town hall points show the municipal number of thyroid cancer cases. The most likely cluster, which is shown with the hatch diagonal stroke in the map, contains 8 municipalities. The numbers of observed and expected cases are 42 and 29.76. The relative risk is 1.411 and the statistical testing p-value against the null hypothesis of geographically homogeneous risk is 0.75. The map in this figure was created with ArcGIS version 10.5 (http://desktop.arcgis.com/).

As there were 115 diagnosed as thyroid cancer cases among 295,032 examinees at the primary examination for the study (See Methods for the details), the standardised municipality-level prevalence ratios in the 59 municipalities in Fukushima Prefecture showed quite high variability, mainly due to the small number of cases in each municipality (Fig. 2). We applied flexibly shaped scan statistics (Flexscan)^21^ to the dataset, resulting in the most likely clusters around the middle part of the prefecture to the west of the FNPP. However, the high p-value (0.758) associated with the cluster meant that even if the risk of the disease was geographically homogeneous, such a degree of regional excess of prevalence is likely to have occurred by chance.

### Detecting general clustering tendency

**Figure 3:**
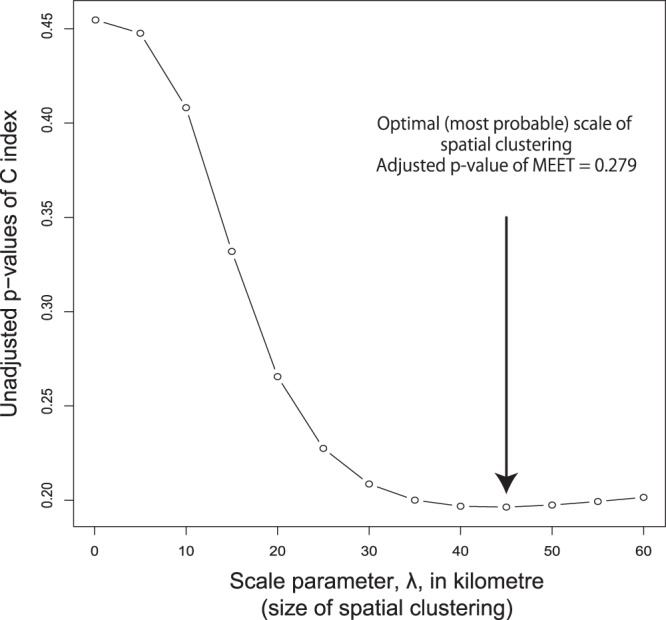
Scale profile of Tango’s spatial clustering test index (C-index) and adjusted p-value of maximised excess events test (MEET).

We also employed Tango’s maximised excess events test (MEET)^22^, a general test of spatial clustering. No clustering tendency was detected in regional thyroid cancer prevalence at all spatial scales tested. Figure 3 provides the profile of C-index at different spatial scales, indicating that 45 km attained the lowest p-value. This indicates that municipal prevalence rates tend to be similar between regions closer than 45 km. The scale is similar to the maximum distance of 49.20 km that exists between town hall locations within the most likely cluster detected by Flexscan. However, the multiple-testing adjusted p-value of MEET was 0.279, which does not support spatial clustering tendency.

### Poisson regression for ecological association with regional indicators

With univariate Poisson regression, no significant relationship was detected between thyroid cancer prevalence and selected regional indicators, including distance from the FNPP, the estimated external radiation dose, altitude, and several census-based indicators (Table 1). The lowest p-value was 0.177 for unemployment. According to the Akaike Information Criterion (AIC), each model using a regional explanatory variable shown in Table 1 did not show any improvement in model fitting against the null model, assuming homogeneous regional risk. Table 2 shows the results of models using the quartile categories of each explanatory variable, considering the possibility of non-linear relationships between the response and explanatory variables. The models using quartile categories did not show any improvement in model fitting against either the corresponding model with a continuous explanatory variable or the null model. No significant trend was identified in the coefficients of the quartile categories for all explanatory variables. The lowest p-value of trend tests for coefficients of the quartile categories was 0.370 for distance from the FNPP.

**Table 1:** Poisson regression results using untransformed explanatory variables.

Variable name	unit	Exp (coefficient of explanatory variable)		p-value of Wald test	Residual Deviance	AIC
		Estimate	95% CI			
(Null model)	NA	NA		NA	46.852	126.92
Proportion of estimated external radiation dose ≥1 mSv	proportion among surveyed people	1.041	(0.616, 1.758)	0.882	46.830	128.89
Distance from the FNPP	1 km	0.997	(0.988, 1.006)	0.503	46.399	128.46
Altitude	100 m	1.078	(0.944, 1.231)	0.269	45.649	127.71
Population density	1000 persons per square kilometre	1.243	(0.274, 5.647)	0.778	46.773	128.84
Proportion of workers in agriculture, forestry and fisheries industries	proportion among workers	0.979	(0.939, 1.021)	0.317	45.800	127.86
Unemployment	proportion among labour force	9.823 × 10^4^	(0.006, 1.749 × 10^12^)	0.177	45.025	127.09
Proportion of professional and technical workers	proportion among workers	3.091	(0.001, 7.773 × 10^3^)	0.778	46.772	128.84

Each row represents a univariate Poisson regression model using one explanatory variable shown in the 1^st^ column (only the null model does not have any explanatory variable). Estimates of intercept terms were omitted. n = 59 (municipalities) for all of the models.

If the p-value of Wald test is small, it indicates that the coefficient is considerably different from zero. AIC, Akaike information criterion; FNPP, Fukushima Daiichi Nuclear Power Plant; N/A, not applicable; CI, confidence interval.

**Table 2:** Poisson regression results using quartile categories of explanatory variables.

Variable name	unit		Exp (coefficient of explanatory variable)		P-value of Wald test (P-value for trend)	Residual Deviance	AIC
			estimate	95% CI			
Proportion of estimated external radiation does ≥ 1 mSv	proportion among surveyed people	Q1	Reference		(0.757)	46.554	132.62
		Q2	1.169	(0.584, 2.340)	0.659		
		Q3	1.059	(0.508, 2.211)	0.878		
		Q4	1.149	(0.602, 2.196)	0.673		
Distance from the FNPP	1 km	Q1	Reference		(0.370)	45.924	131.99
		Q2	0.958	(0.631, 1.454)	0.840		
		Q3	0.945	(0.537, 1.664)	0.845		
		Q4	0.622	(0.221, 1.747)	0.368		
Altitude	100 m	Q1	Reference		(0.623)	43.624	129.69
		Q2	1.439	(0.964, 2.149)	0.075		
		Q3	1.183	(0.612, 2.286)	0.618		
		Q4	1.299	(0.612, 2.756)	0.495		
Population density	1000 persons per square kilometre	Q1	Reference		(0.560)	45.536	131.60
		Q2	1.159	(0.257, 5.227)	0.848		
		Q3	1.653	(0.390, 7.011)	0.495		
		Q4	1.357	(0.333, 5.520)	0.670		
Proportion of workers in agriculture, forestry, and fisheries industry	proportion among workers	Q1	Reference		(0.970)	44.080	130.14
		Q2	0.889	(0.546, 1.448)	0.638		
		Q3	0.547	(0.239, 1.252)	0.153		
		Q4	1.198	(0.486, 2.953)	0.695		
Unemployment	proportion among labour force	Q1	Reference		(0.371)	43.628	129.69
		Q2	0.944	(0.328, 2.722)	0.916		
		Q3	1.351	(0.488, 3.745)	0.562		
		Q4	1.428	(0.510, 4.001)	0.498		
Proportion of professional and technical workers	proportion among workers	Q1	Reference		(0.995)	45.785	131.85
		Q2	1.239	(0.346, 4.441)	0.742		
		Q3	0.880	(0.256, 3.021)	0.839		
		Q4	1.125	(0.356, 3.559)	0.841		

Each estimation result of Poisson regression model using quartile categories (Q1, Q2, Q3, and Q4) of one explanatory variable (shown in the 1^st^ column) consists of four rows to report the estimated coefficients of the four quartile categories. For each model, the lowest quartile (Q1) is set as the reference category. Estimates of intercept terms were omitted. n = 59 (municipalities) for all of the models.

A small Wald test p-value indicates that the coefficient is considerably different from that of the reference category. AIC, Akaike information criterion; FNPP, Fukushima Daiichi Nuclear Power Plant; N/A, not applicable; CI, confidence interval.

### Sensitivity analysis of the undiagnosed positive cases on the spatial analysis

**Figure 4:**
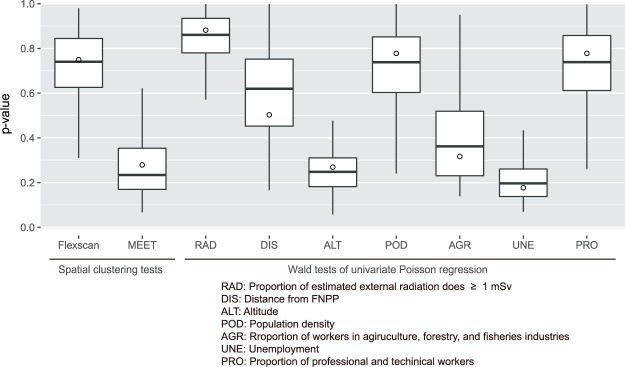
Result of sensitivity analysis of the effects of undiagnosed positive examinees at primary examination on p-values of the spatial analysis. Box-plots were drawn by 100 Monte Carlo simulation runs for p-value of the most likely cluster computed by flexibly shaped spatial scan statistics, the adjusted p-value of maximised excess events test (MEET), and Wald tests of univariate Poisson regression about the coefficient of the explanatory variable. The white circles represent the p-values obtained from the analysis not considering the effects of undiagnosed positive examinees (the same numbers reported in Figs 2 and 3 and Table 1).

The simulation generated additional hypothetical thyroid cancer cases among 195 undiagnosed positive examinees at the ultrasound examination; its median was 11 and inter-quartile range was 4. For all statistical analyses employed, the median of the simulated p-values using the simulated data largely remained unchanged (Fig. 4). Although there is no strict rule about the decision of the alpha level used for the sensitivity analysis, the smallest p-values for each simulation were larger than 0.05 and the simulated distribution of p-values indicated that the judgement of the significance testing employed was unlikely to be changed due to the increase in unreported cases among the undiagnosed cases.

## Discussion

This study showed no substantial geographical clustering in thyroid cancer prevalence among children and adolescent examinees in the first-round of TUE in Fukushima Prefecture. The number of thyroid cancer cases from the programme has often been reported by stressing the unexpectedly large number, suggesting radiation-induced excess cases. The total number of diagnosed cases, however, has been also suspected as a product of over-diagnosis by the mass screening with cutting-edge examination techniques^23^ as in the case of screening-induced “epidemic” of thyroid cancer in South Korea^24,25^. While assessing the validity of the over-diagnosis hypothesis is beyond the scope of our analytical study, assessing the geographical variation in thyroid cancer prevalence provides an alternative approach to determining if the ‘epidemic’ is real. A measurable and notable geographical variation in radiation dose is expected in the environment within Fukushima Prefecture; therefore, the detection of excess risks in radiation contamination areas has gained attention.

It is important to note that the cluster detection techniques (the spatial scan statistics and MEET) used in this study, are both categorised as ‘general tests’^26^, which do not assume predefined focus on clusters, such as around the nuclear plant, and provide conservative testing results, by considering the multiple testing procedures about positions and scales, of possible clusters. This choice of analysis is justified because of the difficulty in estimating the distribution of internal and external radiation exposure in the target population, due to the short half-life of radioactive iodine-131 (approximately 8 days). Due to the no-clustering tendency from the two general test results, it is hard to believe that there are unknown spatial clusters showing increases in thyroid cancer incidence by unknown radiation exposure distribution at the municipality level.

If we had a suspicious source of contamination, ‘focused tests’^26^ evaluating distance trends in prevalence from the source location would be another form of cluster detection with higher power. The Poisson regression model using the distance from the FNPP and the proportion of external radiation exposure of more than 1 mSv for the first four months after the FNPP accident, can be considered as a class of simple focused tests. It should be noted that the distribution of thyroid doses from radioactive iodine-131 could differ from that from external doses, and the uncertainty of the distributional information about thyroid doses from radioactive iodine is one of the limitations of this study. While the distribution of external exposure shows a clear north-western directional trend from the FNPP, the contaminated areas with radioactive iodine is estimated to be relatively circular since a plume with radioactive iodine flew in a southern direction from the FNPP in an earlier phase of the accident^4^. The distance from the FNPP was thus also worth assessing. Similar to previous studies^10–13^, we found no association between the standardised prevalence ratio and both of the regional indicators focusing on the FNPP location.

To estimate the background risk distribution of childhood and adolescent thyroid cancer (apart from exposures to the radiation from the FNPP accident), we tested the association between altitude and regional socio-economic indicators. Our finding in Fukushima Prefecture does not correspond to the increase in thyroid cancer incidence with higher altitude as previously shown in Italy^18^. Although altitude was considered a surrogate indicator of iodine deficient dietary habits, this relationship would not hold because Japan is generally an iodine-rich area and the Japanese diet is high in seafood. Tsubokura *et al*.^27^ surveyed the variation in iodine deficiency among children in Fukushima Prefecture after the disaster in 2011. They showed a relatively higher risk of iodine deficiency in coastal areas, which was possibly due to the restriction of inshore fishing in the region; however, no extreme iodine deficiency occurred in the entire area of the prefecture. We suspect that the rate of iodine deficiency among children was low even in the mountainous parts of the region.

We also found no association between the standardised prevalence of thyroid cancer and urban-rural indices and socio-economic indicators of municipalities. An increase in thyroid cancer incidence with higher areal SES was reported in previous studies^19,20^. A plausible explanation for this was higher areal SES reflecting better access to healthcare, leading to higher detection rates of the disease. However, this previous finding was not supported in the present study, possibly because of the overall high coverage rate of ultrasound examination.

The results of a series of spatial statistics-based analyses employed here indicated neither substantial spatial clusters nor clustering tendency of the sex- and age-standardised prevalence rate of thyroid cancer at the municipality level. According to our simulation-based sensitivity test, the possible increase in thyroid cancer cases among positive ultrasound examinees without confirmation testing did not alter the findings of spatial analysis.

Our study has several limitations. Firstly, approximately 18.3% of the target population (about 6.71 thousand), mostly adolescents, did not undergo the primary examination^28^. The rate of non-participation at the primary examination was highest (about 32.5%) in the Aizu region of the prefecture, which is the most western part of the prefecture and estimated as the least radiation-contaminated zone, while the lowest (about 12.5%) in the 13 municipalities with evacuation zone. Due to the geographical tendency of non-participation, a cluster of high prevalence rates could be more likely to be detected in more contaminated areas^8^, but we did not observe this during our study period. Although we adjusted for sex and age effects of the examinees at the primary examination, there remains the possibility that this situation reduced the size and caused unknown biases in the distributional characteristics of diagnosed cases. However, we did not conduct a sensitivity test on this mainly due to the lack of adequate information about the non-examinees, in particular regarding whether they resided in the registered municipality when the disaster occurred in March 2011.

Secondly, even if the guidelines for testing were unchanged during the study period, the actual practices of testing may have differed at different time periods after the disaster, depending on differences in the residents’ anxiety about radiation exposure in different regions. Among approximately 2,000 persons who underwent confirmatory testing, the biopsy rate to obtain cytological diagnosis was the highest (47.7%) in the 13 municipalities with evacuation zone, possibly reflecting patients’ high anxiety about radiation contamination; this rate was the lowest (16.7%) in the Aizu region^28^. Similar to the argument concerning rates of non-participation, although there is a possibility that the difference in biopsy rates could induce overestimation of diagnosed thyroid cancer in the most contaminated region, we did not observe excess prevalence rates in that region during our study period. However, further careful consideration is needed to determine possible causes of biases regarding regional prevalence rates of thyroid cancer.

Thirdly, since there were numerous possibilities when constructing regional indicators, there is the possibility of identifying a meaningful regional predictor of thyroid cancer prevalence in future. Furthermore, since we could not manipulate the definition of the basic spatial units, our analysis was dependent on the current municipalities. We might have failed to detect smaller clusters of the disease within a municipality, although such small-scale analysis could suffer from the small number of cases more seriously compared to the analysis at the municipality level.

Fourthly, our study design is ecological, and individual factors, apart from age and sex, were not assessed for standardisation of prevalence. Further studies are needed that include more individual factors such as family history concerning the disease and lifestyle (including dietary habits, obesity and overweight, and radiation exposure including medical use)^29^ as well as exposure to radioactive iodine. Finally, we did not consider the temporal difference in the primary examination. It has been pointed out that the age-at-exposure distribution of cases in the first round TUE shows increasing risk at older ages (i.e., exposed as adolescents) while the distribution for those cases occurring after the CNPP accident shows the opposite pattern, a sharp increase in youngest ages at exposure. This difference indicates that the age distribution of cases in the first round TUE follows the normal increase in incidence of the disease with age^30^. Ohira *et al*. found no contribution of the primary examination date to being diagnosed with thyroid cancer at the individual level in the first round of TUE^12^.

While our results indicate that the thyroid cancer cases detected through ultrasound examination in the first four years after the FNPP accident are unlikely to be attributable to regional factors, including radiation exposure resulting from the FNPP accident, further studies are needed to consider longer-term health outcomes of the accident in future rounds of TUE^31^.

## Methods

### Study subjects

A total of 367,649 children and adolescents (<19 years of age at the time of the accident) were registered as the target population in the first round of TUE according to the latest summary report of the program^28^. The 59 municipalities at the primary examination were allocated by fiscal year: 2011 for the evacuation zone comprising 13 municipalities near the FNPP, 2012 for 12 municipalities in the middle part of the prefecture, and 2013 for the remaining regions including the most inland part, Aizu, and coastal regions except the evacuation zone (34 municipalities). Since there were late examinees, the first-round examination included 300,473 persons who underwent primary examination from October 2011 to April 2015. When the primary examination using ultrasonography found nodules (size 5.1 mm or more) or cysts (size 20.1 mm or more) or identified the urgent need for confirmatory testing due to clinical reasons, confirmatory examinations were recommended. Confirmatory testing included a detailed ultrasound, blood testing, urine analysis, and fine-needle aspiration cytology (FNAC) if needed. Details of the survey protocol are provided elsewhere^16^.

The Fukushima Medical University carried out the ultrasound examination programme and compiled the surveillance database which was the data source for this study. The dataset as of 27 February 2017 included 299,908 persons who underwent the primary examination. We excluded 1,523 persons who did not live in Fukushima Prefecture when the earthquake occurred on 11 March 2011. It should be noted that the excluded population included one thyroid cancer case. We further excluded 3,353 persons who did not provide information on residential municipality on the day of the earthquake. Consequently, data on the 295,032 subjects who underwent primary examination were analysed in this study. Among 2,246 examinees who tested positive in the primary examination, 2,051 (91.4%) underwent confirmatory testing. There were 115 cases that were classified as malignant or highly suspicious of malignancy based on FNAC at confirmatory testing. We regarded these cases as diagnosed thyroid cancer cases for this study. These cases were aggregated by municipality for this analysis. According to the summary report of TUE^28^, the malignancy rate after surgery for the FNAC-based diagnosed thyroid cancer cases was 99.0% for the first round TUE (102 FNAC-based diagnosed cases received surgery, and of those, 101 cases were post-surgically confirmed with thyroid carcinoma).

This study was approved by the Ethics Committee of the Fukushima Medical University (approval no. 1318). Written informed consent for the study was obtained from the parents of every participant. All methods were carried out in accordance with the applicable guidelines and regulations for the use and analysis of the Fukushima Health Management Survey data, managed by the Radiation Medical Science Center, Fukushima Medical University.

### Spatial statistical analysis

Since age and sex are the most established determinants of thyroid cancer^32^, we used the sex and age-standardised prevalence ratio at the municipality level as follows:

$$sp{r}_{i}={o}_{i}/{e}_{i},$$

where *i* is the index of the municipality, *o*_*i*_ is the number of observed cases of thyroid cancer and *e*_*i*_ is the sex- and age-adjusted expected number of cases, also defined as:

$${e}_{i}=\sum _{k}po{p}_{ik}p{r}_{k},$$

where *k* is the index of sex (male and female) and 5-year age group (0–4, 5–9, 10–14, 15–19, and 20 or older), *pop*_*ik*_ is the number of primary ultrasound examinees of the *k*th group in the *i*th municipality, and *pr*_*k*_ is the prefectural marginal prevalence rate of the *k*th group. The age groups were based on the age of the examinees when they underwent the primary examination. We assume that the geographic distributions of observed cases, {*o*_*i*_}, were independently and randomly generated by the Poisson distribution with the expected number of cases, {*e*_*i*_}:

$${o}_{i} \sim Poisson[{r}_{i}{e}_{i}],$$

where *r*_*i*_ is the relative risk of thyroid cancer in the *i*th municipality. The null hypothesis of our spatial analysis is the situation of geographically homogeneous risk (*r*_*i*_ = 1 for all *i*). In other words, the hypothesis means that the prevalence rates of thyroid cancer are only determined by the age and sex distribution of examinees.

### Method 1: detecting locally elevated risk regions

An alternative hypothesis based on the above null hypothesis is that there were regions with higher risks compared to the others. Kulldorff^15^ proposed an algorithm using a circular scan window which exhaustively searches for elevated risk regions among the set of all possible centre locations and sizes of circle window. The method uses likelihood ratio statistics by comparing risks inside and outside a circular window region. The window region with the highest likelihood ratio is called the ‘most likely cluster’. A Monte Carlo simulation under the assumption of homogeneous risk provides the null distribution of the statistics by which we may test the significance of the most likely cluster. Since the test statistic is defined by only one value in the entire study region, this method avoids the problem of multiple testing.

Tango and Takahashi^21^ proposed Flexscan to search for irregularly-shaped clusters with elevated risk. In this method, scanning windows are defined as a set of topologically connected spatial units (sharing a border point or line). If the true cluster is non-circular (e.g. linear), the modified spatial scan statistic is likely to have stronger power compared to the traditional circular scanning. Radiation dose is distributed in a non-circular form because radioactive plumes from the FNPP were carried by the wind; areas with high doses stretched from the FNPP in a north-westerly direction and then moved southward in the most northern part of the prefecture. Thus, if the distribution of standardised prevalence ratio is associated with radiation exposure, we expect that the flexibly shaped scan statistic is more likely to detect the elevated risk regions compared to the traditional circular scanning. The results may highlight specific regions with high risk on the map. For the computation, we used Flexscan version 3.1.2. For the technical details on this method, see Supplementary Note.

### Method 2: detecting general clustering tendency

A tendency of clustering of prevalence can be considered as a positive spatial autocorrelation of risks. Detection of such a tendency may indicate that the risk of thyroid cancer has a spatial structure reflecting the distributions of some geographic factors. Tango’s MEET^22^ is considered the method with the highest power for detecting general tendency of spatial clustering^33^.

The test has two phases. Firstly, it measures the C-index of clustering tendency which requires a scale parameter, which determines “closeness” in geographic space in advance. Although the C-index can be used for statistical testing, the result depends on the choice of scale parameter. Searching for the optimal (most probable) scale of spatial clustering by testing different scale parameter series results in the multiple comparisons/testing. The second phase of MEET involves computing the adjusted p-value of clustering tendency with a predefined set of possible scale parameters and Monte Carlo simulation evaluation, by considering the statistical uncertainty of optimal scale selection. For the technical details, see Supplementary Note. We ran the R script of MEET^26^ in the R version 3.4 environment (The R Core Development Team, Vienna, Austria).

### Method 3: Poisson regression

Ecological regression is another approach to assessing whether regional relative risks are geographically structured, particularly for those which have geographical indicators of suspicious factors of diseases under investigation. Poisson regression is a common choice based on the same assumption of Poisson process for disease clustering tests. A univariate Poisson regression using the log link function is:

$${o}_{i}\sim Poisson[{r}_{i}{e}_{i}],$$

$${\rm{l}}{\rm{n}}({r}_{i})={\beta }_{0}+{\beta }_{1}{x}_{i},$$

where *x*_*i*_ is the explanatory variable in the *i*th municipality. We considered seven municipal indicators as explanatory variables: distance from the FNPP, the estimated external radiation dose, altitude, and several census-based socio-economic indicators.

Using focused tests which are designed to evaluate clustering around a pre-fixed geographic object, we selected the distance from the FNPP and the proportion of estimated external radiation doses of equal to or more than 1 mSv during the first four months after the accident at the FNPP. The distance variable was measured as the Euclidean distance from the FNPP to the town hall of each municipality. The information on estimated effective exposure to radiation from the FNPP was based on individual behavioural data that were collected as part of the Fukushima Health Management Survey. Following the methods of Ohira *et al*.^12,13^ who used the same information for defining regions with different radiation dose levels, we defined the variable, external radiation exposure, as a surrogate collective indicator of thyroid equivalent dose.

Related to iodine deficiency is altitude, which is a known geographic risk indicator for thyroid cancer, as it is an indicator of low access to iodine-rich food, such as fish and seaweed^18^. We employed the altitude of the town hall locations as the representative value for each municipality.

Furthermore, we included 2010 census-based indicators. In several previous studies, rural-urban indices and areal SES indicators were prepared to capture the general characteristics of thyroid cancer geographic distribution^19,20,34,35^. Since there were no established rural-urban indicators in Japan, we included population density as a simple surrogate of urbanicity^36^, and the proportion of agriculture, forestry, and fisheries industries workers as a rurality indicator. Regarding areal SES, the rate of unemployment in the entire labour force was included as the most influential component of areal deprivation^36^. Finally, the proportion of professional workers was used as an index of affluence.

For every explanatory variable, we applied the untransformed values as well as the quartile categories for examining possible non-linear relationships. For the computation of Poisson regression, we used the “glm” function in the R version 3.4 environment. To measure the distance and the cartographic mapping, we used ArcMap 10.5 (Environmental Systems Research Institute, Redlands, CA, USA). Euclidean distances were measured on the basis of map coordinates under rectangular plane (zone 9) projection with the Japanese Geodetic Datum 2011.

### Sensitivity analysis of the undiagnosed positive cases on the spatial analysis

To evaluate the uncertainty caused by the 195 undiagnosed positive examinees, we conducted a simulation-based sensitivity analysis for each method as follows:

Step 1: From those who accepted to undergo confirmatory testing, we computed the rates of diagnosed thyroid cancer cases among the positive examinees at primary examination by sex and 5-year age groups for the entire study region. We assumed that the rate was determined by sex and age, with no regional differences in the rates between positive examinees of the primary examination who accepted or declined to undergo confirmatory testing.

Step 2: Applying the binomial probability of being diagnosed cases with the rate computed above for each group of undiagnosed examinees by sex and age groups, we simulated the number of diagnosed cases among undiagnosed examinees for each municipality using random number generation. By adding them to the observed number of cases, we created a hypothetical dataset if every positive examinee underwent confirmatory testing.

Step 3: Using the simulated number of cases, we conducted the same analyses for cluster detection and Poisson regression.

Step 4: We repeated steps 2 and 3 for *T* times to obtain the distribution of statistical testing results, such as p-values.

We implemented this sensitivity analysis for flexibly shaped scan statistics, MEET, and Poisson regression using R scripts with *T* = 100.

## Electronic supplementary material

Supplementary Note Technical details of applied methods

## Data Availability

The Radiation Medical Science Centre (RMSC) of Fukushima Medical University authorised us to analyse the current data of thyroid examinations from the Fukushima Health Management Survey (FHMS) in this study. The centre currently restricts usage of FHMS data to members or observers of special committees of the FHMS. T.N., K.T. and H.T. are observers in such special committees. The data are confidential and are not publicly available.
