# Supplementary material for: Spatial analysis of the geographical distribution of thyroid cancer cases from the first-round thyroid ultrasound examination in Fukushima Prefecture
Source: Sci Rep. 2018 Dec 5;8:17661. doi: 10.1038/s41598-018-35971-7 (PMC6281575; doi:10.1038/s41598-018-35971-7)
Supplement: Supplementary file 1 — Supplementary Note Technical details of applied methods [file 41598_2018_35971_MOESM1_ESM.docx]

**Spatial analysis of the geographical distribution of thyroid cancer cases from the first-round thyroid ultrasound examination in Fukushima Prefecture**

Authors:

Tomoki Nakaya, PhD,

Kunihiko Takahashi, PhD

Hideto Takahashi, PhD

Seiji Yasumura, MD, PhD

Tetsuya Ohira, MD. PhD

Hitoshi Ohto, MD, PhD

Akira Ohtsuru, MD, PhD

Sanae Midorikawa, MD, PhD

Shinichi Suzuki, MD, PhD

Hiroki Shimura, MD, PhD

Shunichi Yamashita, MD, PhD

Koichi Tanigawa, MD, PhD

Kenji Kamiya, MD, PhD

# **Supplementary information**

## Supplementary Note Technical details of applied methods

### Flexibly shaped spatial scan statistics (Flexscan)

Let $L_{w}$ be the likelihood ratio function based on Poisson distribution for window *w,* expressed as:

$$L_{w}=\left( \frac{o_{i}}{e_{i}} \right)^{oi}\left( \frac{\sum_{i} o_{i}-c_{i}}{\sum_{i} o_{i}-e_{i}} \right)^{\left( \sum_{i} o_{i}-c_{i} \right)}I(c_{i}>e_{i})$$

where *I* is the indicator function which is one in case of $c_{i}>e_{i}$ or zero otherwise. The test statistic, *S* is

$$S=\max_{w} L_{w}$$

When the value of *S* is higher than the critical value obtained from the simulated null distribution (assuming that the given numbers of cases, randomly distributed, are only dependent on the distribution of expected sizes of cases), the null hypothesis is rejected.

Flexscan searches exhaustively for all the combinations of connected regions for each focusing unit, with the maximum length of topological connection. The default size is set at 15. The number of Monte-Carlo simulations run was set at 9,999.

### Maximized Excess Events Test (MEET)

The method introduces an index of spatial clustering, *C*, defined by:

$$C\left( \lambda\right)=\sum_{i,j} \left( o_{i}-e_{i} \right)\left( o_{j}-e_{j} \right)\exp\left( -4\frac{d_{ij}^{2}}{\lambda^{2}} \right)$$

where $d_{ij}$ is the distance between the municipalities *i* and *j*. The distance is calculated by the Euclidean distance between town hall locations on 11 March 2011. The parameter, *λ*, controls the spatial scale of clustering to be detected. A smaller value is sensitive to small-scale spatial clustering while a larger value is more sensitive to larger-scale clustering. MEET provides the adjusted p-value of the statistics to take into account the multiple testing about the scale parameter. The adjusted p-value is defined as:

$$P_{min} =\min_{0<\lambda\leq\lambda_{max}} Pr\left\{ C(\lambda)>c(\lambda)|H_{0},\lambda\right\}$$

where *c* is the observed value of *C*. For practical implementation, 'line search' for the optimal lambda is applied for predefined discrete series of *λ*, and the probability of the testing is obtained, again by Monte-Carlo simulation. Considering that Tango suggested max $d_{ij}$/4 as the upper limit of line search^15^, we considered 13 values of *λ* = {0.1, 5, 10, 15, …, 50, 55, 60}. The number of Monte-Carlo simulation runs was set as 9,999.
